# New score tests for age-at-onset linkage analysis in general pedigrees

**DOI:** 10.1186/1753-6561-3-s7-s97

**Published:** 2009-12-15

**Authors:** Andrea Callegaro, Hae-Won Uh, Quinta Helmer, Jeanine J Houwing-Duistermaat

**Affiliations:** 1 Department of Medical Statistics and Bioinformatics, Leiden University Medical Center, Leiden, The Netherlands Medical Center, PO Box 9600, 2300RC Leiden, The Netherlands

## Abstract

Our aim is to develop methods for mapping genes related to age at onset in general pedigrees. We propose two score tests, one derived from a gamma frailty model with pairwise likelihood and one derived from a log-normal frailty model with approximated likelihood around the null random effect. The score statistics are weighted nonparametric linkage statistics, with weights depending on the age at onset. These tests are correct under the null hypothesis irrespective of the weight used. They are simple, robust, computationally fast, and can be applied to large, complex pedigrees. We apply these methods to simulated data and to the Genetic Analysis Workshop 16 Framingham Heart Study data set. We investigate the time to the first of three events: hard coronary heart disease, diabetes, or death from any cause. We use a two-step procedure. In the first step, we estimate the population parameters under the null hypothesis of no linkage. In the second step, we apply the score tests, using the population parameters estimated in the first step.

## Background

It is well known that heterogeneity results in loss of statistical power when studying genetic factors of complex genetic diseases. To deal with heterogeneity additional data such as covariates (e.g., age at onset, known genetic factors) are collected. In this paper we are interested in adjusting linkage for age at onset.

Frailty models have been proposed for age-at-onset linkage analysis [1-5]. Gamma frailty models are particularly attractive because the gamma-distributed random effect can be easily integrated out and it allows the use of observable marginal survival functions [1-4]. A drawback of these models is that their corresponding likelihood becomes very complex for large pedigrees. To solve this problem, we propose a score test based on a composite likelihood [6].

A second model for multivariate survival data is the log-normal frailty model. Using this model, Pankratz et al. [5] proposed a likelihood-ratio approach for linkage. In the spirit of Lebrec and Houwelingen [7], we derive a robust and simpler score test, using an approximation of the likelihood around the null random effect.

## Methods

### Gamma frailty model: pairwise likelihood approach

Let *T*_*ij *_be the random variable of age at onset for relative *j *in family *i*, *i *= 1, ..., *N*. Let (*t*_*ij*_, *d*_*ij*_) be the observed data where *t*_*ij *_is the observed age at onset if *d*_*ij *_= 1 and age at censoring if *d*_*ij *_= 0. The conditional hazard for individual *j *in family *i*, with covariates *x*_*ij *_and random effect *Z*_*ij*_, is given by *λ*(*t*_*ij *_| *x*_*ij*_, *Z*_*ij*_) = *λ*_0_(*t*_*ij *_| *x*_*ij*_)*Z*_*ij*_. Without loss of generality, we assume that *E [Z] *= 1. The baseline hazard *λ*_0_(*t*) is the hazard for *x *= 0 and *Z *= 1. The frailty *Z *is decomposed into the sum of independent gamma distributed effects, namely a linkage effect, a residual additive effect, and a non-shared environment effect. The scale parameter is common to all of the effects and is defined as the sum of the shape parameters. When the proportion of alleles shared identically by descent (IBD) for a relative pair (*j*, *k*) is known (*π*_*jk*_), the marginal bivariate survival function can be derived from the additive gamma frailty model [4]. The bivariate survival function depends on the marginal survival functions, on the variance of the random effect (*σ*_*G*_^2^), and on the pairwise correlation. The correlation *ρ*_*jk*_(*π*_*jk*_) = (*π*_*jk*_-*Eπ*_*jk*_)*γ *+ *ρ*_*jk *_depends on the IBD through the linkage parameter *γ*. Under the null hypothesis (*H*_0_:*γ *= *γ*_0 _= 0), the correlation is equal to the correlation in the population (*ρ*_*jk*_). The marginal correlation between the *i*^th ^and the *j*^th ^individual is a function of their expected proportion of alleles shared IBD, *ρ*_*jk *_= *a*^2^*Eπ*_*jk*_, where *a*^2 ^is the portion of the variance explained by the total additive effect.

We use a retrospective likelihood [4] and, in order to deal with general pedigrees, we consider a pairwise likelihood approach [6]. For *N *families, the corresponding score statistic is a weighted nonparametric linkage (NPL) statistic

Here, elements of the weight matrix *W *are given by , where  is the prospective bivariate likelihood. The operator *vec*(*A*) places the *n *columns of the *m *× *n *matrix *A *into a vector of *mn *× 1. In the case of uncertain IBD status, the variance of the proportion of allele shared IBD () can be estimated by simulations. Note that the classical mean IBD test is a weighted NPL statistic [Eq. (1)] with weight equal to *W*_*jk *_= *d*_*j *_× *d*_*k*_.

### Log-normal frailty model

Let *d*, Λ_0_, and *V *= log *Z *be the *n*-dimensional vectors of the disease status, the baseline cumulative hazards at the observed age, and the normally distributed random effects of the *n *members of a particular pedigree, respectively. The random effect *V *follows a multivariate normal distribution with mean zero, and variance-covariance matrix Σ with elements Σ_*jk *_= *σ*_*N*_^2^*ρ*_*jk*_(*π*_*jk*_). The log-likelihood can be approximated by using a second-order Taylor approximation around *V *= 0. For small random effects and known baseline cumulative hazard, the vector of standardized martingale residuals behaves as a normal distribution. Integrating over the distribution of the random effect gives *M *= (*d*-Λ_0_)/Λ_0_ ~ *N*(0, Σ_1_), where Σ_1_ = Σ + *diag*(1/Λ_0_). The score statistic derived from the retrospective likelihood is a weighted NPL statistic [Eq. (1)] with weight matrix *W *= Σ_1_^-1^*M*(Σ_1_^-1^*M*)'-Σ_1_^-1 ^and Σ_1 _taken in *γ *= 0. In this paper we approximate the baseline cumulative hazard with the marginal cumulative hazard.

## Materials

### Estimation of the population parameters

Three phenotype files were provided: Original Cohort participants, Offspring participants, and Generation 3 participants. We combined the three files and used this dataset as a random sample from the population. The total number of individuals considered was 6879. The number of disease-free survival events was 644 (248 coronary heart diseases, 385 diabetes, and 98 deaths), with prevalence around 10%. We estimated the marginal survival functions stratified by sex using the Kaplan-Meier estimator. By age 60 years, 20% of males and 10% of females were affected. Using these estimated survival functions we fitted a marginal pairwise correlated gamma frailty model. The sib-sib marginal correlation was *ρ *= 0.46 and the variance estimated by the gamma frailty models was  = 0.93. The sib-sib marginal correlation was *ρ *= 0.5 and the variance estimated by a log-normal frailty model [5] was  = 0.43.

### Pedigree data preparation

In the Genetic Analysis Workshop (GAW) 16 Framingham Heart Study (FHS) data 765 pedigrees with 2 to 301 genotyped subjects were available. To simplify the IBD computation, large pedigrees were split into *n *= 1599 nuclear families. The number of nuclear families with at least one affected sibling was *n *= 488. Only 46 nuclear families were available with at least two affected siblings.

### Single-nucleotide polymorphism (SNP) data selection

The GAW16 Framingham dataset included 550 k SNP genotype data. Using the nuclear families with at least one affected individual (2275 individuals), we selected 15 k SNPs informative for linkage. First, markers with known physical position were selected (497 k). Second, 10 markers per centimorgan with minor allele frequency larger than 0.15 were considered (37 k). Finally, SNPs were simulated on 250 sib-pairs in order to select 15 k SNPs with the highest information content. The information content of the final set of SNP was around 85%.

### Simulated data

**Figure 1:**
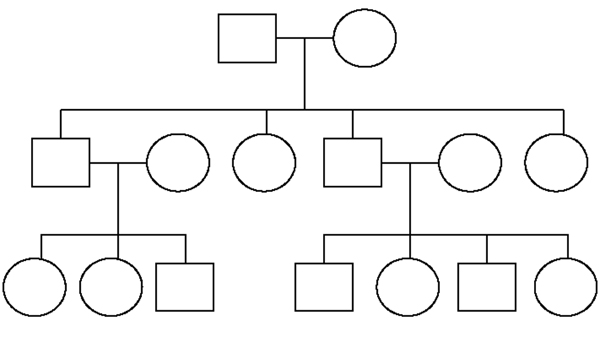
**Pedigree structure of 15 individuals used for simulating data**.

To assess power and type I error rates, we simulated data using a frailty model with parameter values estimated in the GAW16 FHS data. The random effect was gamma-distributed with a mean of one and variance of  = 0.93. The baseline hazard was derived from the marginal hazard. The random effect was decomposed into the sum of three components: one locus-additive genetic effect (explaining 60% of the variability), one shared environmental effect (explaining 20% of the variability), and one unshared environmental effect. We simulated pedigrees with 15 members (Figure 1). Marker data were simulated far from any disease locus (null hypothesis) and close to the disease locus, which explains all the additive genetic variance (alternative hypothesis).

## Results

### Simulated data results

Table 1 shows the type I error rates based on 5000 replications and the power based on 1000 simulations, for sample size of 300 families with at least two affected siblings. On simulated data, the proposed methods have correct type I error rates. For our simulation settings, taking into account age at onset considerably increases the power to detect linkage. On a moderately sized pedigrees (15 members), the log-normal approach is more powerful than the pairwise gamma frailty approach.

**Table 1:** Estimates of type I error rates and power

	Null hypothesis		Alternative hypothesis	
		
Method	*α *= 0.05	*α *= 0.01	*α *= 0.05	*α *= 0.01
Mean IBD	0.05	0.01	0.34	0.14
Gamma	0.05	0.01	0.94	0.80
Log-normal	0.05	0.01	0.98	0.85

### Application to the FHS dataset

**Figure 2:**
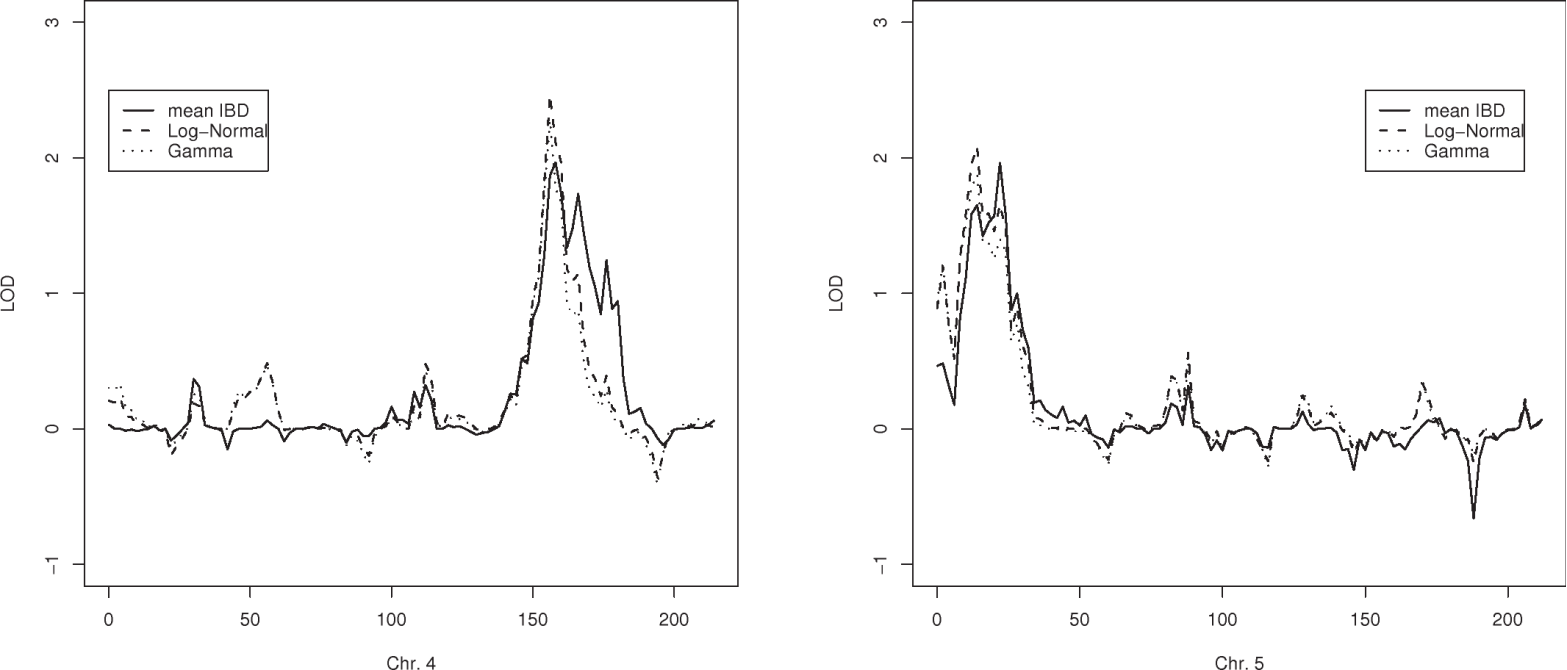
**Age at onset genetic linkage analysis of GAW16 FHS dataset**. LOD scores on chromosomes 4 (left) and on chromosome 5 (right).

We performed a genome-wide linkage analysis using the unweighted NPL test (mean IBD test) with variance of the allele shared IBD estimated by simulations [8]. Figure 2 shows the two highest LOD scores (close to LOD = 2), which are located on chromosomes 4 and 5, respectively.

We applied the proposed methods to the data of these two chromosomes. The linkage analysis was performed on all the nuclear families (*n *= 1599), on the families with at least one affected siblings (*n *= 448) and on the subset of families with at least two affected siblings (*n *= 45). The maximum LOD-scores were obtained considering only families with at least two affected siblings. Figure 2 shows the results on this subset of families. On chromosome 4, adjusting for age at onset increases the maximum LOD score from 2 to 2.5. On chromosome 5, with the proposed methods the maximum LOD score is in a slightly different location (10 cM) with respect to the unweighted mean IBD test (25 cM). Results on chromosome 5 are replicated on the larger set of families with at least one affected sibling (data not shown).

## Discussion

In this paper we proposed two approaches for age-at-onset linkage analysis in general pedigrees. We applied the proposed methods to the GAW16 FHS data in two suggestive regions identified by the standard NPL method. The maximum LOD-scores were obtained analyzing only the set of families with at least two affected siblings. This can be due to the fact that affected individuals carry most of the information for linkage. On the densest pedigrees, adjusting for age at onset slightly increased the evidence for linkage. However, it is difficult to interpret the results because of the small number of events.

Because GAW16 FHS families were randomly selected, it was possible to estimate the marginal information directly from the data. When marginal information is known from previous twin (family) studies, the proposed methods can be applied to ascertained families.

For the two identified regions, association analysis in the presence of linkage may be the next step. The proposed models can be easily extended to study association in the presence of linkage by including the genotype of the siblings as a covariate.

In this paper we computed IBD probabilities using MERLIN and we estimated the variance of the allele shared IBD using simulations [8]. Because this software can deal only with small to moderately large families, we split large families into nuclear families. An alternative approach is to estimate IBD probabilities using Markov-chain Monte Carlo methods, which now provide this information for general pedigrees. Sampled inheritance vectors can also be used to estimate the variance of the allele shared IBD in the denominator of the score statistic.

Software to apply the proposed methods is freely available [9].

## Conclusion

We proposed two new score tests for age of onset linkage analysis. Both methods are simple and can be applied to general pedigrees. Simulations showed that the proposed methods outperform the traditional affected-only NPL method. On the application to the GAW16 FHS data, adjusting for age at onset slightly increased the interesting linkage peaks.

## List of abbreviations used

FHS: Framingham Heart Study; GAW: Genetic Analysis Workshop; IBD: Identical by descent; NPL: Nonparametric linkage; SNP: Single-nucleotide polymorphism

## Competing interests

The authors declare that they have no competing interests.

## Authors' contributions

AC participated in method development, carried out data analysis, and drafted the manuscript. QH carried out the SNP data selection. JJH-D participated in method development. H-WU acquired the data. All authors read, critiqued, and approved the final manuscript.
